# Bacteria isolated from the cuticle of plant-parasitic nematodes attached to and antagonized the root-knot nematode *Meloidogyne hapla*

**DOI:** 10.1038/s41598-019-47942-7

**Published:** 2019-08-07

**Authors:** Olivera Topalović, Ahmed Elhady, Johannes Hallmann, Katja R. Richert-Pöggeler, Holger Heuer

**Affiliations:** 1 Institute for Epidemiology and Pathogen Diagnostics, Julius Kühn-Institut — Federal Research Centre for Cultivated Plants, Braunschweig, Germany; 2 Department of Plant Protection, Faculty of Agriculture, Benha University, Benha, Egypt

**Keywords:** Agroecology, Symbiosis, Zoology

## Abstract

Plant-parasitic nematodes are associated with specifically attached soil bacteria. To investigate these bacteria, we employed culture-dependent methods to isolate a representative set of strains from the cuticle of the infective stage (J2) of the root-knot nematode *Meloidogyne hapla* in different soils. The bacteria with the highest affinity to attach to J2 belonged to the genera *Microbacterium*, *Sphingopyxis*, *Brevundimonas*, *Acinetobacter*, and *Micrococcus* as revealed by 16S rRNA gene sequencing. Dynamics of the attachment of two strains showed fast adhesion in less than two hours, and interspecific competition for attachment sites. Isolates from the cuticle of *M*. *hapla* J2 attached to the lesion nematode *Pratylenchus penetrans*, and *vice versa*, suggesting similar attachment sites on both species. Removal of the surface coat by treatment of J2 with the cationic detergent CTAB reduced bacterial attachment, but did not prevent it. Some of the best attaching bacteria impaired *M*. *hapla* performance *in vitro* by significantly affecting J2 mortality, J2 motility and egg hatch. Most of the tested bacterial attachers significantly reduced the invasion of J2 into tomato roots, suggesting their beneficial role in soil suppressiveness against *M*. *hapla*.

## Introduction

Plant-parasitic nematodes (PPN) impose a global threat for food security. The severity of the crop damage caused by PPN depends on the nematode feeding strategy, their initial population density, the existence/absence of an antagonistic supremacy in soil and the defense of the plant. In terms of economic costs and research focus the genus *Meloidogyne* (root-knot nematodes, RKN) is a leading PPN genus^1^. It is characterized by the formation of typical galls or root-knots on roots of the infected plants. A remarkable stage in the life cycle of the RKN is the second-stage juvenile (J2) which is the only stage that has the capacity to move through soil and infect plants. On the way to the roots, J2 interact with a vast range of microbes that cohabit in a specific soil type. The soils where the nematode performance is impaired by the native microflora are referred to as suppressive. There are many examples showing that the nematode suppression is removed by soil sterilization or by biocide and biofumigation treatments^2–5^. Recent studies have shown that a very specific subset of microorganisms attaches to the cuticle of infective stages of PPN in soil^2,6^. However, these interactions were only studied by culture-independent methods in order to detect the species composition of nematode-attached microbial communities. Studies on the ability of the attached soil microbiota to antagonize nematodes, for which cultured isolates are needed, are still scarce. From investigations on the attachment of spores of the gram-positive bacterium *Pasteuria* sp. to PPN^7–10^, it was suggested that the carbohydrate residues of the glycoproteins on the nematode surface coat bind lectins from the bacterial surface in a very specific manner. The nematode surface coat is a glycoprotein layer that overlays the cuticle. It is most probably secreted by the hypodermis^11,12^, although there are some suggestions that it originates from the excretory or nervous system^13^. In contrast to the immobile and rigid cuticle that is shed off in an ecdysis events at the end of each juvenile stage, the surface coat is a very dynamic structure with a continuous turn-over and renewal^10,11,14^. For instance, it has been shown that the pre-incubation of J2 of *Meloidogyne javanica* in detergents reduced the binding of human red blood cells to the nematode surface. The binding of the human blood cells was recovered after 20 hours^15^, suggesting a rapid change of the nematode surface due to formation of a new surface coat. The surface coat may play a pivotal role in the specific attachment of soil microbes to infective stages of PPN^8,9,16–19^.

In the current study, we established the methods to isolate and identify bacteria that attach to J2 of the northern RKN species *Meloidogyne hapla* in different soils, and studied the ability of attached bacterial isolates to antagonize nematodes. More specifically, we investigated the effects of attached bacteria on the motility, mortality, and on the invasion into tomato roots of J2, as well as effects of the bacteria on hatching of eggs. To elucidate the role of the surface coat in the attachment of vegetative cells of soil bacteria to the nematode surface, we studied if the surface treatment of J2 with cationic and anionic detergents affect the attachment rate. The dynamics of bacterial attachment over the incubation time and competition for attachment sites were investigated. To investigate if the attachment sites are conserved among two species of the order Tylenchida, it was tested whether bacteria isolated from the surface of *M*. *hapla* better attach to their original host than to *Pratylenchus penetrans*, and *vice versa*. We believe that this study is a step forward in understanding the interactions between PPN and the bacteria that they encounter in soil. It gave evidence that the nematode-attached microbiome plays a significant role in soil suppressiveness against PPN.

## Results

### Isolation and identification of bacterial attachers to J2 of *M*. *hapla* or *P*. *penetrans* in different soils

J2 of *M*. *hapla* or *P*. *penetrans* were baited in suspensions of diverse agricultural soils, recovered, intensively washed with sterile water, and plated on culture media to isolate nematode-attached bacteria. Among the isolates, fourteen strains from *M*. *hapla* and three strains from *P*. *penetrans* were selected for further analysis based on unique genomic BOX fingerprints. Their taxonomic affiliation was determined by 16S rRNA gene sequencing (Supplementary Table S1). A very high specificity in bacterial attachment to nematodes was correlated with a low diversity of the identified strains in different soils. The highest number of the bacterial CFU that attached to *M*. *hapla* J2 was found for the genus *Microbacterium* for which six different strains were isolated from J2 (i.10, i.20, i.44, i.47, K6, i.14).

### Attachment rates of the bacterial isolates to J2 of *M*. *hapla*

**Figure 1:**
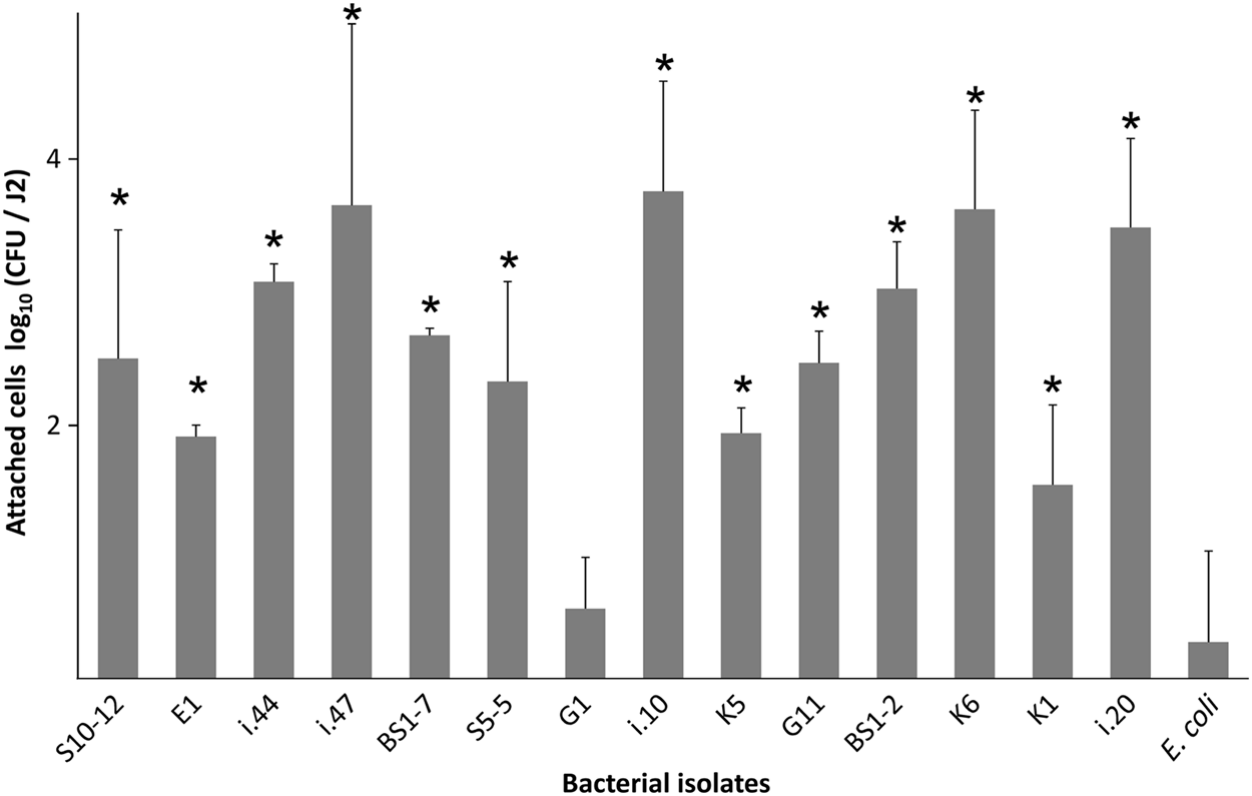
Attachment of bacterial strains to J2 of *Meloidogyne hapla*. Bacterial strains originating from the surface of *M*. *hapla* J2 were incubated overnight with J2 and the number of attached CFU was determined by plating of J2 on agar media. Genera of bacterial strains: *Microbacterium* (K6, i.10, i.20, i.44, i.47), *Sphingopyxis* (BS1-2, G11), *Brevundimonas* (BS1-7, S5-5), *Acinetobacter* (E1, S10-12), *Micrococcus* (K5), *Staphylococcus* (K1), *Kocuria* (G1). *E*. *coli* EK5-23 served as a negative control for unspecific attachment, as this strain was not isolated from nematodes. Stars indicate significant differences to the control (Dunnett test, n = 3). Error bars represent standard deviations of log-transformed CFU counts.

To confirm that the bacteria isolated from the J2 indeed highly attach to the cuticle of J2 of *M*. *hapla*, we screened 15 isolates for their re-attachment to the J2 surface. All the tested bacterial strains, except G1, attached with a significantly higher number of cells to J2 than the negative control *E*. *coli* EK5-23 (Fig. 1). The highest densities of attached cells were observed for the isolates K6, i.10, i.20, i.44, i.47 (*Microbacterium* spp.), BS1-2, G11 (*Sphingopyxis* spp.), BS1-7 (*Brevundimonas* sp.), and S10-12 (*Acinetobacter* sp.). Strains K6, i.10 and i.47 of the genus *Microbacterium* had 4200 to 5700 CFU per J2. For strains i.20, i.44, BS-1-2, G11, BS1-7, and S10-12, more than 300 attached CFU per J2 were observed. Isolates K5 (*Micrococcus* sp.), S5-5 (*Brevundimonas* sp.), and K1 (*Staphylococcus* sp.) had 40 to 200 attached CFU per J2. The attachment of the strain G1 (*Kocuria* sp.) did not significantly differ from the control *E*. *coli* EK5-23, both having less than 10 CFU per J2.

### Comparison of bacterial attachment to *M*. *hapla* and *P*. *penetrans*

**Figure 2:**
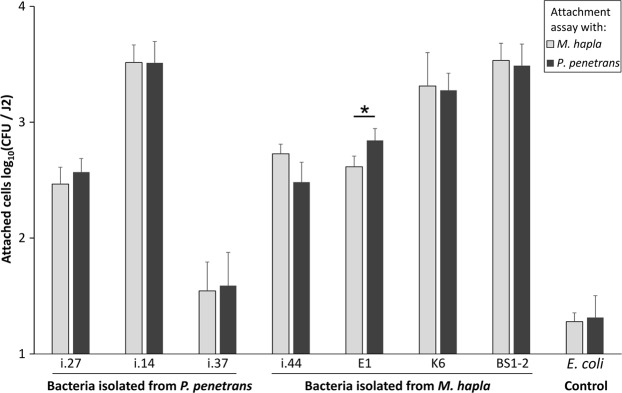
Attachment of bacterial strains isolated from the cuticle of *Pratylenchus penetrans* or *Meloidogyne hapla* to *M*. *hapla* and *P*. *penetrans*. The tested isolates were i.27 (*Pseudomonas* sp.), i.37 (*Alcaligenes* sp.), i.14, i.44, K6 (*Microbacterium* spp.), E1 (*Acinetobacter* sp.), and BS1-2 (*Sphingopyxis* sp.). The assay with *E*. *coli* EK5-23 served as a control. Error bars represent standard deviations of log-transformed CFU counts. The star indicates a significant difference in the attachment of isolate E1 to *M*. *hapla* and *P*. *penetrans* (Tukey’s HSD test, n = 3).

In order to see whether the bacterial isolates that were originally isolated from the cuticle of J2 of *M*. *hapla* would differ in the attachment to J2 of *P*. *penetrans*, and *vice versa*, we tested several *M*. *hapla*- (i.44, K6, BS1-2, E1), and *P*. *penetrans*-originated isolates (i.14, i.27, i.37). In general, there was a good attachment of the tested isolates to both nematode species in comparison to the negative control *E*. *coli* EK5-23 (Fig. 2). The lowest number of attached CFU was observed for the isolate i.37 that originated from *P*. *penetrans*. However, no significant differences were found in the attachment of this isolate to *M*. *hapla* and *P*. *penetrans*. The latter also applied to the remaining two *P*. *penetrans*-originated isolates, i.14 and i.27, and for *M*. *hapla*-originated isolates BS1-2 and K6. The hypothesized nematode species dependency on the bacterial attachment was found only for the isolate E1 (*Acinetobacter* sp.) that was isolated from *M*. *hapla* J2. However, the attachment of the isolate E1 had a higher degree of binding to *P*. *penetrans*. Overall, these results indicated that the bacterial attachment to *M*. *hapla* and *P*. *penetrans* was more dependent on the bacterial strain than on the nematode species, and that the binding affinity of the tested isolates was generally very high, with a mean attachment of up to 3000 CFU per J2.

### Dynamics of bacterial attachment to *M*. *hapla*, and competition for attachment sites

**Figure 3:**
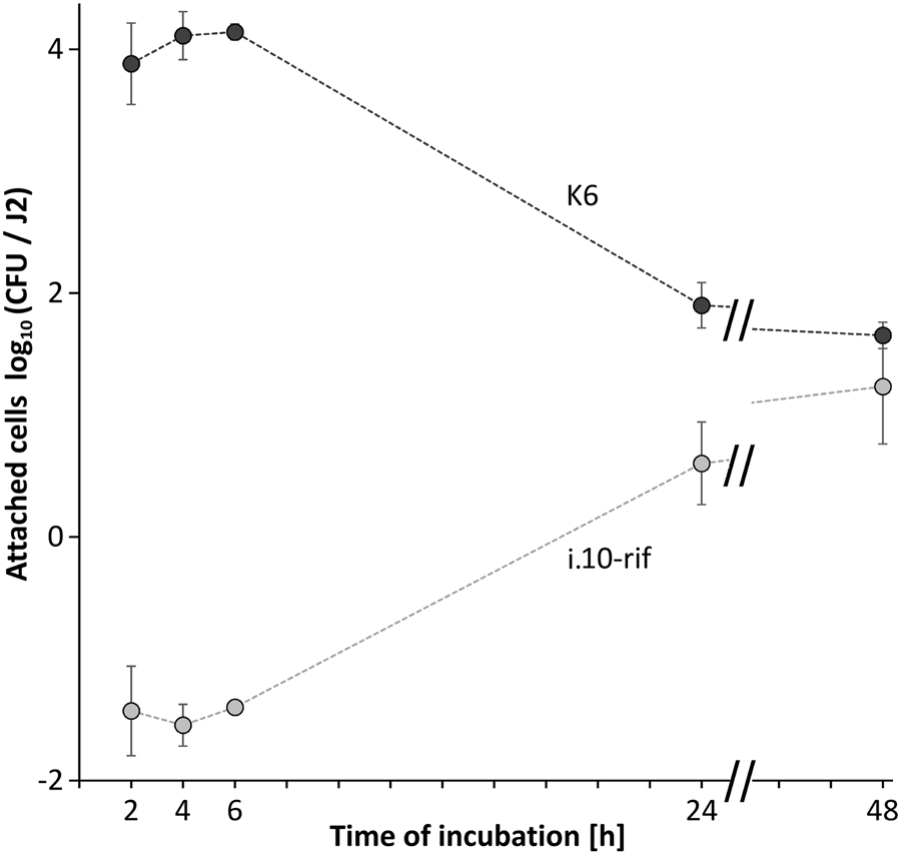
Kinetics of the competitive attachment of the isolates K6 and i.10-rif (*Microbacterium* spp.) to J2 of *Meloidogyne hapla*. Both strains together were incubated with *M*. *hapla* J2. The number of attached bacterial cells per J2 was determined at 2, 4, 6, 24 and 48 hrs. Error bars represent standard deviations (n = 4).

To test if the attachment rate changes over time, J2 of *M*. *hapla* were incubated in a suspension of the bacterial isolate K6 and sampled at different time points. We selected a rifampicin-resistant mutant of the isolate i.10 that showed a high attachment rate to *M*. *hapla* J2. The strain i.10-rif was added together with the isolate K6 to each replicate tube at a 100-fold lower density. We had the intention to quantify it as an internal reference by plating on a rifampicin-containing medium. The attachment of the isolate K6 occurred fast in the first two hours, and the number of attached CFU slightly increased over the next four hours (Fig. 3). The CFU number of the strain K6 on the nematode’s surface significantly dropped after 24 and 48 hours of incubation. Concomitantly, the attachment of the reference isolate i.10-rif had the opposite trend. The number of attached cells of the strain i.10-rif was at least 1000-fold lower than expected from the ratio of K6 and i.10-rif in the suspension. After 24 and 48 hours the number of attached cells of i.10-rif significantly increased, and was not significantly different from K6 after 48 hours.

**Figure 4:**
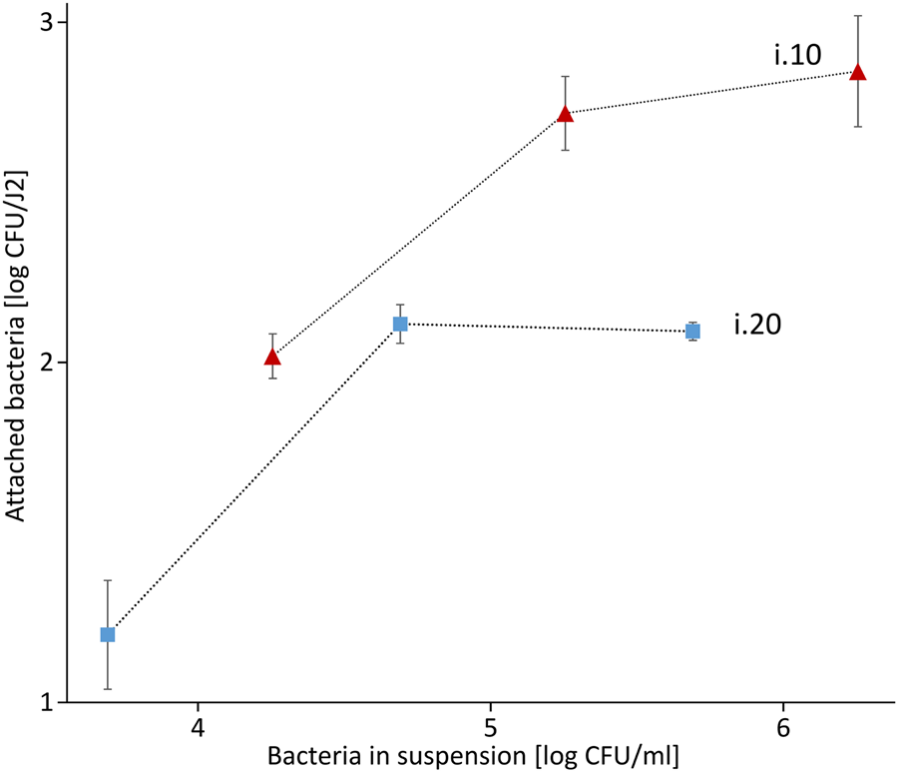
The attachment rate to J2 of *M*. *hapla* in different concentrations of bacterial cell suspensions of the isolates i.10 and i.20 (*Microbacterium* spp.). J2 of *M*. *hapla* were incubated in 100 µl of different concentrations of the bacterial isolates i.10 (10^6^, 10^5^ and 10^4^ CFU) and i.20 (10^5^, 10^4^ and 10^3^ CFU) and the number of attached bacterial cells per nematode was determined. Error bars represent standard deviations (n = 3).

Additionally, we tested how three different concentrations of the bacterial isolates i.10 and i.20 affect the attachment rate to J2 of *M*. *hapla* (Fig. 4). In case of both bacterial isolates, the attachment exponentially enhanced with the increase of bacterial concentration from 10^4^ to 10^5^ CFU/ml (i.10), and from 10^3^ to 10^4^/ml (i.20). However, further increase in bacterial concentration did not or did very little enhance the number of attached bacterial CFU per J2 for both tested isolates, suggesting that the nematode’s cuticle was already saturated with bacterial cells.

### Removal of the surface coat of *M*. *hapla* J2 affected the attachment rate

**Figure 5:**
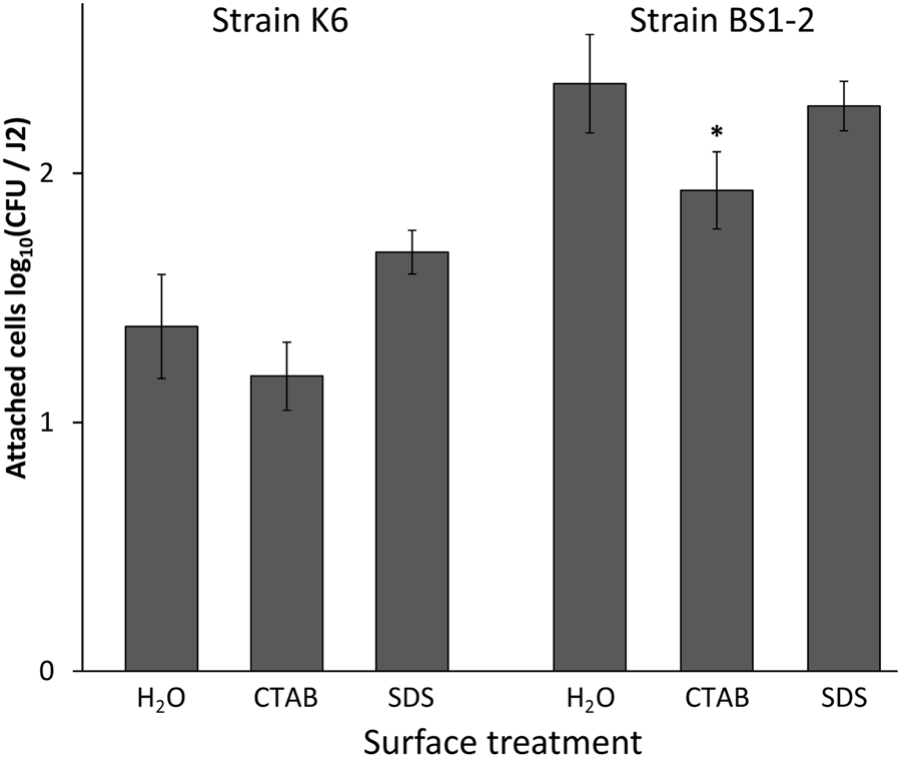
Effect of surface coat removal by detergents on the attachment of bacterial isolates K6 (*Microbacterium* sp.) and BS1-2 (*Sphingopyxis* sp.) to *Meloidogyne hapla*. The J2 were treated with 0.25% CTAB, 0.25% SDS or sterile water before the attachment assay with bacterial isolates K6 or BS1-2. The number of attached bacterial cells was determined after an overnight J2 incubation in bacterial cell suspensions. Error bars represent standard deviations. The star represents a significant treatment effect of CTAB compared to SDS and water (Tukey’s HSD test, n = 3).

Despite inconsistency in previous studies on the removal of the nematode surface coat, there are indications that some anionic and cationic detergents strip the antigens from the nematode surface coat and reduce bacterial binding^15,20,21^. In order to test whether the surface coat is an active component of bacterial attachment to the nematode cuticle we treated nematodes with anionic (SDS) and cationic (CTAB) detergent prior to their incubation in bacterial cell suspensions of the isolates K6 (Gram positive, *Microbacterium* sp.) and BS1-2 (Gram negative, *Sphingopyxis* sp.). The treatment of *M*. *hapla* J2 with the cationic detergent CTAB reduced the bacterial binding in comparison to the control that was only treated with sterile tap water (STW) (Fig. 5). More specifically, the mean attachment was significantly reduced after the treatment with CTAB in comparison to the treatment with STW for the isolate BS1-2. There was a trend of reduction by CTAB for the isolate K6 that was not statistically supported. The treatment with SDS had no significant effect on the attachment of the isolates in comparison to the treatment with STW. It is worthy to notice that although there is a significant reduction in the bacterial attachment to J2 after the treatment with CTAB in comparison to the STW control or SDS treatment, the attachment was still considerably high for both bacterial isolates. This suggested a minor effect of the surface coat on the bacterial attachment in the short term.

### Effects of attached bacteria on J2 mortality, J2 motility, and egg hatch

**Figure 6:**
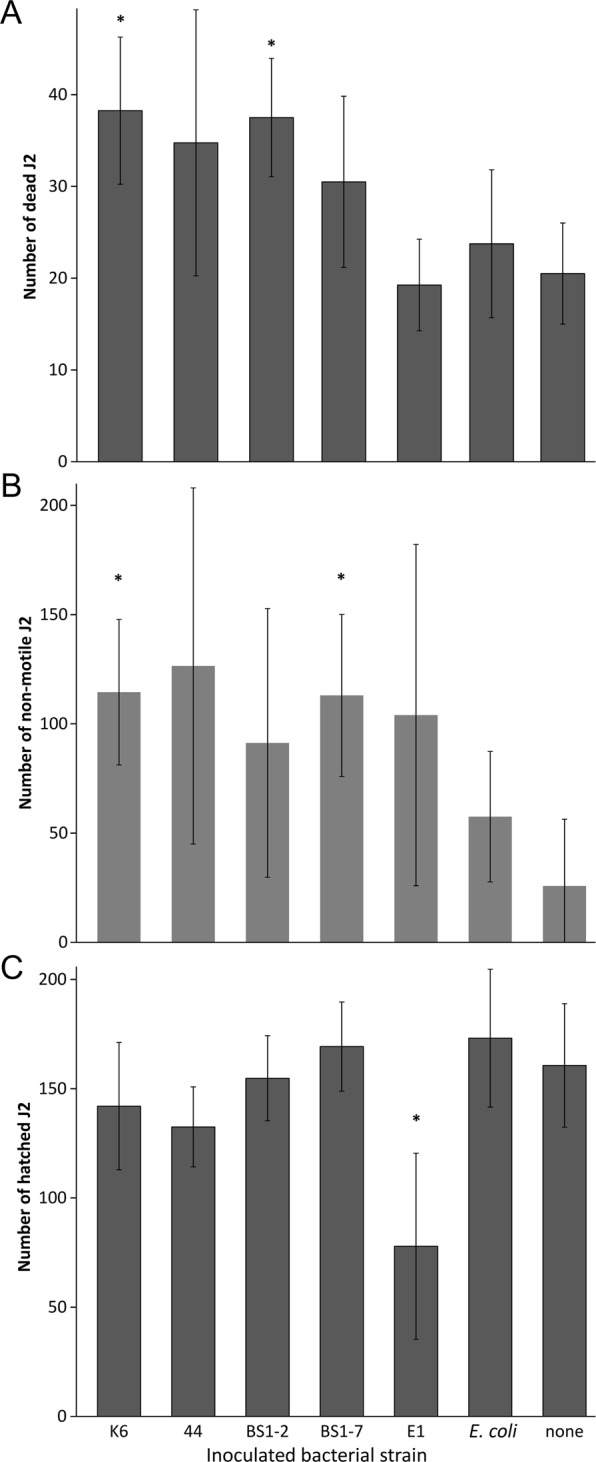
Effect of attached bacteria on mortality, motility and hatching of *Meloidogyne hapla* J2. The bacterial isolates and the control *E*. *coli* EK5-23 were attached to J2, and the effects on J2 mortality (**A**), J2 motility (**B**), and egg hatch (**C**) were determined, in comparison to J2 incubated with sterile water. Error bars represent standard deviations. The isolates that were tested were K6 (*Microbacterium* sp.), BS1-2 (*Sphingopyxis* sp.), i.44 (*Microbacterium* sp.), BS1-7 (*Brevundimonas* sp.), and E1 (*Acinetobacter* sp.). Stars indicate significant differences to the control without bacteria (Dunnett test, n = 4).

To test whether the bacterial isolates that showed a high attachment to J2 of *M*. *hapla* express an antagonistic potential against nematode performance *in vitro*, we designed experiments on their effects on J2 motility, J2 mortality and egg hatch. In particular, the bacterial cell suspension of the isolates K6 (*Microbacterium* sp.) and BS1-2 (*Sphingopyxis* sp.) almost halved the number of alive J2 in comparison to the treatment with STW during a 3-day incubation period (Fig. 6A). The bacterial cell suspension of the isolates i.44, BS1-7, E1 and the J2-non-associated *E*. *coli* EK5-23 did not significantly affect J2 mortality in comparison to the STW control. Isolates K6 and BS1-7 significantly impaired J2 movements compared to the STW control (Fig. 6B). In suspensions of the strains i.44, BS1-2 and E1 the mean number of non-motile J2 also had a higher trend than in the controls. In suspensions of *E*. *coli* EK5-23, the average J2 motility was the highest and did not significantly differ from the STW control.

After a 5-day incubation of eggs of *M*. *hapla* in the respective bacterial suspensions at room temperature, only isolate E1 caused a significant reduction in hatching rate of J2 compared to the STW control (Fig. 6C). The average number of hatched J2 in a suspension of the isolate E1 was 78 ± 43, which is about half less than in the STW control.

### Specific bacterial attachers reduced *M*. *hapla* J2 invasion into the roots

**Figure 7:**
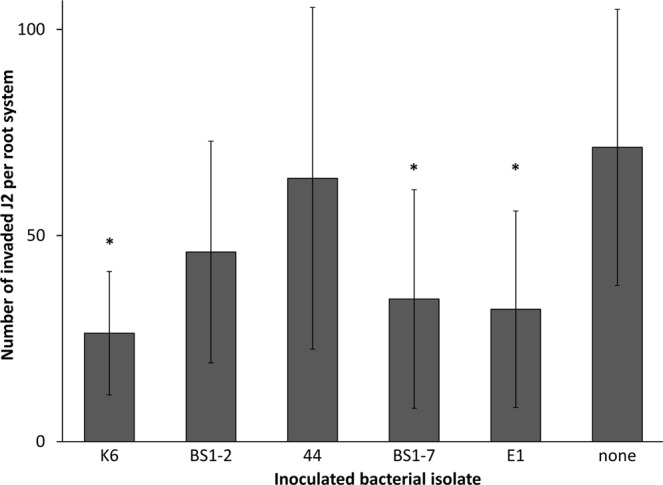
Effect of bacterial isolates on invasion of *Meloidogyne hapla* J2 into tomato roots. Bacterial isolates from the cuticle of *M*. *hapla*, the control strain *E*. *coli* EK5-23, or sterile water were inoculated to the rhizosphere of tomato plants five days before inoculation of *M*. *hapla* J2. The isolates that were used were K6 (*Microbacterium* sp.), BS1-2 (*Sphingopyxis* sp.), i.44 (*Microbacterium* sp.), BS1-7 (*Brevundimonas* sp.), and E1 (*Acinetobacter* sp.). The number of invaded J2 was determined after root staining with acid fuchsin. Stars indicate significant differences to the control without inoculated bacteria (Dunnett test, n = 10). Error bars represent standard deviations.

A greenhouse set-up was used to further characterize the biological effect of the attached isolates against the invasion of *M*. *hapla* J2 into tomato roots. The rhizosphere of tomato plants was drenched with bacterial cells of the respective isolates five days prior to J2 inoculation. In comparison to the control, where the plants were challenged with STW, three bacterial isolates caused a significant reduction of J2 invasion into the roots at 7 dpi, namely K6, BS1-7 and E1. On average, 26 J2 were present inside the root treated with the isolate K6, which is almost three times less than in the control treatment. The isolates BS1-2 and i.44 did not significantly reduce J2 invasion into the roots compared to the control but showed a trend of reduction (Fig. 7).

### Microscopic visualization of attached *Microbacterium* sp. K6 on J2 of *M*. *hapla*

**Figure 8:**
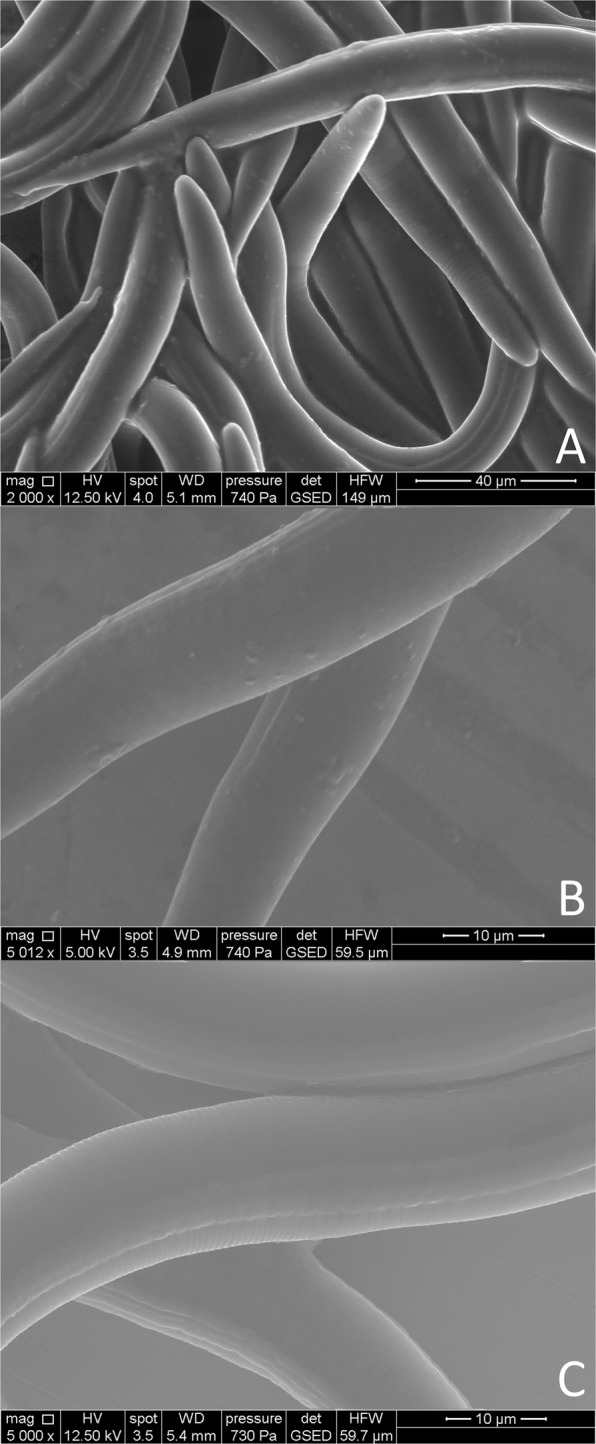
Environmental scanning electron microscopy of J2 of *Meloidogyne hapla* with or without attached bacterial cells. The J2 with attached bacterial isolate K6 (*Microbacterium* sp.) were photographed after 24 h of incubation (**A**,**B**). As control, J2 were incubated in sterile tab water (**C**).

The single bacterial cells of the isolate K6 attaching to the cuticle of *M*. *hapla* J2 were visualized using ESEM (Fig. 8). The figures show an abundant attachment of the isolate K6 after 24 h of incubation with the J2. The attachment seemed to be random and not specific for certain body regions of the nematode. In comparison, no bacterial cells were visible on the nematode’s surface after incubation in STW. Light microscopy showed the bacterial cells sometimes attached in groups, and we suppose that this was influenced by the bacterial behavior of being densely packed together in water.

## Discussion

We employed culture-dependent methods to isolate nematode-associated soil bacteria and discovered that J2 of *M*. *hapla* recruit a very specific subset of bacterial community from different soils. In fact, it was previously shown by next generation sequencing that the number of soil microbiome attached to the nematode’s cuticle is low and that the binding occurs independently of their relative abundance in soil^6^. In the re-attachment experiments all the tested bacterial isolates attached well to the cuticle of *M*. *hapla* J2, and these were assigned to the genera *Microbacterium*, *Brevundimonas*, *Acinetobacter*, *Sphingopyxis*, *Micrococcus*, and *Staphylococcus*. All these genera have already been associated to PPN. *Microbacterium* sp. has been reported in association to the cyst nematode *Heterodera schachtii* in the fields infested with this PPN species^22^. In addition *Microbacterium nematophilum* was found to induce morphological changes in the rectal and post-anal region of the nematode *Caenorhabditis elegans*^23,24^, and the presence of glycoconjugates in the nematode cuticle seems to play role in the adhesion^25^. *Acinetobacter* sp. was found attached to the cuticle of the PPN *M*. *hapla*, *M*. *incognita* and *P*. *penetrans*^2,6^. It has also been reported to impose a nematicidal activity against *C*. *elegans*^26^. Although *Brevundimonas* sp., *Sphingopyxis* sp., *Micrococcus* sp. and *Staphylococcus* sp. were found associated to PPN^2,6,27,28^, their role in nematode suppression has not been studied more extensively. Importantly, some of the identified *M*. *hapla* J2-attached bacterial genera are also recognized as plant growth promoting rhizobacteria. In particular, a combination of *Microbacterium* sp. and *Bacillus* sp. strains enhanced the growth of apple trees^29^. *Brevundimonas* sp. and *Acinetobacter* sp. are also included in the group of plant growth promoting rhizobacteria according to some authors^30,31^. However, it should be noted that both plant growth promoting function and the ability to antagonize different nematode species and populations is highly species- and strain-specific within the respective bacterial genera. Further studies should be conducted in order to reveal the association of bacterial strains obtained in this study and plant growth promotion.

The surface coat of nematodes is considered to play an important role in the interaction events between nematodes and other organisms^7–9,15,32^. A dynamic nature and a constant turnover of the surface coat affect the bacterial presence on the nematode’s cuticle^11^. Several scientists attempted to track the changes in the bacterial attachment to nematodes after incubating nematodes in different detergents for the purpose of the surface coat removal^13,20,33^. In this study, we found that the treatment of *M*. *hapla* J2 with cationic detergent CTAB significantly reduced the subsequent attachment of one of the bacterial isolates to the nematode surface, as opposed to the treatment with anionic detergent SDS. However, this did not seem to be of biological relevance since the nematode surface was still saturated with bacterial cells. There is a certain controversy in the literature regarding the success of detaching the surface coat components from the nematode’s body using detergents. It was pointed out that cationic detergents, in contrast to the anionic ones, are much more successful in this regard due to the net negative charge of the nematode surface coat^21^. This was consistent with the study of Pritchard *et al*.^33^ on the removal of the surface antigens from the animal parasitic nematode *Nematospiroides dubius* using CTAB. However, Spiegel *et al*.^34^ tested the attachment of *Pasteuria* spores to *M*. *javanica* after exposure of J2 to 1% SDS for 30 min. Attachment of *Pasteuria* spores immediately after exposing nematodes to SDS was very low (5.5 spores per J2, compared to 20.8 spores per J2 in the water control). This effect was similar when J2 were stored at 4 °C for 2 days after SDS treatment, but not at 25 °C. On the other hand, Davies and Danks^20^ found no effect of SDS treatment on the attachment of spores to the J2 of *M*. *incognita*. When they treated J2 with 1% SDS for 2 hours at room temperature, the attachment of spores was even slightly higher compared to the control. The effect was the same with other detergents they used. The reduced attachment only occurred in case when nematodes were treated with detergents at 100 °C for 2 min, but this high temperature raises the question of nematodes’ viability.

In this study, we also showed the importance of the incubation time in bacterial cell suspension. The attachment of the tested *Microbacterium* sp. isolate K6 was the highest within the first 6 hours of incubation. This was expected as some studies showed that the binding process occurs within several minutes in case of *Pasteuria* sp. and RKN^35^ to 30 min for *Corynebacterium rathayi* and the seed gall nematode *Anguina agrostis*^36^. A steady decrease in the attachment of the isolate K6 at 24 hours of the incubation could be attributed to the fact that the surface coat of nematodes is constantly replaced and renewed^11,21^, therefore the bacterial cells might have been removed together with the surface coat components in the washing centrifugation steps. On the other hand, a slight increase in the attachment of the reference i.10-rif isolate at 24 hours of the incubation may be correlated with the increase of available attachment sites when the K6 isolate detaches from the J2 surface. However, the attachment was still low compared to the K6, suggesting a low initial concentration of this isolate and/or a lower competitiveness for the attachment sites.

The difference in the bacterial attachment to two different PPN species used in this study was rather low. As seen in the results only one isolate originally recognized as an “attacher” to *M*. *hapla* differed in the attachment between the J2 of M. *hapla* and the J2 of *P*. *penetrans*, albeit showing a high degree of attachment in both cases. This was a bit surprising in comparison to the very high specificity of attachment of *Pasteuria* spores to different populations of the same nematode species^37^. Similarly, Elhady *et al*.^6^ found differences in the attachment of both soil bacteria and fungi to the PPN *M*. *incognita* and *P*. *penetrans*. However, in contrast to the latter study we used only the J2 stage of *M*. *hapla* and *P*. *penetrans* to avoid potential differences in the surface coat composition between nematode life stages^10,28,38–40^. Our study suggests that the attachment sites on the nematode cuticle of the J2 are more conserved. Similarly, Davies *et al*.^37^ found a high degree of variation in the attachment of different populations of *Pasteuria* spp. to nematodes, but the attachment of these populations to J2 of *M*. *incognita* and *M*. *arenaria* was not always significantly different.

Out of a vast range of microorganisms present in soil, only a few make a physical interaction with nematodes. Some microorganisms can parasitize nematodes or use them as the carriers to the plant roots. It seems that there is a strong relationship between the way of the feeding strategy of a microorganism and the chemotaxis of nematodes towards them. As exemplified by the attraction of nematodes to the nematophagous fungi, nematodes were most attracted to obligate endoparasitic fungi^41^, and the least to saprophytic fungi^42^. This was independent of the feeding strategy of nematodes. However, it seems that the attachment concept is much more complex for obligate bacterial parasites of PPN^37,38^. Interestingly, in our study bacterial species that attached to J2 of *M*. *hapla* were not obligate parasites of PPN. In *in vitro* assays we demonstrated that some of these bacterial strains exhibit nematicidal and nematistatic effects on juveniles, in particular isolates K6, i.44 (*Microbacterium* spp.), and BS1-7 (*Brevundimonas* sp.). Non-parasitic rhizobacteria antagonize nematodes in two most common ways: (1) by production of metabolic compounds that inhibit hatching and attraction of nematodes to the roots, and (2) by degradation of some root compounds that affect nematode behavior^43^. It was suggested that these bacteria interact with the plant root surface in a lectin-specific manner and that this leads to a recognition of nematodes by plants^43^. In the greenhouse assay our results showed a reduced J2 penetration into the roots when the plants were pretreated with bacterial cell suspensions of the isolates K6 (*Microbacterium* sp.), BS1-7 (*Brevundimonas* sp.), and E1 (*Acinetobacter* sp.). Interestingly, it was reported that volatile organic compounds (VOC) of *Microbacterium* sp. isolated from the egg masses of the RKN *M*. *exigua* on coffee roots caused an extremely high mortality of J2 in *in vitro* assay. In only 3–4 hours of J2 exposure to these volatiles there was more than 50% of dead J2^44^. Cordovez *et al*.^45^ have detected several VOC to prime the growth promotion of *Arabidopsis thaliana*. These VOC were identified as dimethyl disulfide, dimethyl trisulfide, S-methyl 2-methylpropanethioate, and S-methylpentanethioate and four ketones. Dimethyl disulfide is a VOC that was proven to be effective against PPN in several studies^46–48^. In contrast to the isolates K6 and BS1-7, the isolate E1 did not affect behavior of *M*. *hapla* J2 *in vitro*, but was the only isolate that reduced the hatching of J2 from the eggs. It is reported that some bacteria either produce enzymes (e.g. chitinases) or toxins that affect the eggs, or impair hatching factors of the host plants^49^. However, since only J2 were inoculated to the roots in the invasion assay, we assume that the bacterial isolate E1 can trigger induced systemic resistance in plants and prevent J2 invasion. Many examples on the involvement of induced systemic resistance in nematode control have started to emerge^50–57^. We suggest that joint efforts of a direct antagonism and induced systemic resistance in plants are responsible for the reduced J2 invasion into tomato roots in our study.

In conclusion, while PPN move through soil microbes attach to their cuticle in a very specific manner, which means that only a small subset of bacteria evolves a physical interaction with them. Bacterial mutants may shed light on conserved mechanisms required for PPN attachment. We showed that the bacteria that commonly attach to J2 of *M*. *hapla* in different soils were not necessarily obligate parasites of PPN. They belonged to the genera *Microbacterium*, *Sphingopyxis*, *Brevundimonas*, *Acinetobacter*, *Micrococcus*, or *Staphylococcus*. The bacterial attachment to the surface of *M*. *hapla* J2 occurred very fast and the treatment of nematode surface coat with detergents still allowed bacterial cells to attach to a high extent. We also showed that the bacteria that were originally highly attached to *M*. *hapla* J2 had the same tendency to attach to the J2 of *P*. *penetrans*, and *vice versa*. This means that the antagonistic effect of these bacterial isolates could be broadened against more PPN groups. Non-plant-parasitic nematodes should be assayed as well to understand whether this attachment is specifically directed at PPN and thus might belong to a protective microbial shield of plants. The *in vitro* assays indicated a reduced nematode performance when *M*. *hapla* J2 were exposed to cells of such bacteria. This was supported in the greenhouse assay where drenching of tomato plant rhizospheres with the isolates from the genera *Microbacterium*, *Brevundimonas* or *Acinetobacter* reduced J2 invasion into tomato roots seven days after J2 inoculation. Our study suggests that non-parasitic bacteria have a high affinity to attach to the infective stages of PPN in soil and can be considered as important contributors to soil suppressiveness against PPN. However, further studies are needed to see if they persist throughout the nematode life cycle and if they can be cultured from females or eggs.

## Materials and Methods

### Soil baiting experiments with surface-sterilized J2

Eggs of PPN were collected on 20 µm sieves. J2 were allowed to hatch, then exposed to 0.02% HgCl_2_ for 2 min, washed with STW, and incubated for 4 h in an antibiotic solution of 200 mg/l streptomycin sulfate, 25 mg/l rifampicin, and 10x CellGuard (PanReac, AppliChem). After incubation, the J2 were extensively washed on a 5-µm sieve with STW to remove any traces of antibiotic compounds.

Nine field soils were collected from different regions in Germany, namely Geisenheim (G; sandy clay soil with 2.7% humus, pH 7.4, 49°59′01″N, 7°57′25.5″E), Klein Wanzleben (KWS; sandy soil with 6.3% humus, pH 6.9, 52°03′07.2″N, 11°23′13.2″E), Dahnsdorf Schlag 2 (D2; less sandy loam with 1.1% humus, pH 5.9, 52°06′16.1″N, 12°38′40.7″E), Dahnsdorf BS1 (D-BS1; less sandy loam with 1.1% humus, pH 5.8, 52°06′21.9″N, 12°38′13.7″E), Quedlinburg Schlag 5 (S5; less sandy loam with 2.5% humus, pH 6.9, 51°46′9.51″N, 11°9′0.41″E), Quedlinburg Schlag 9 (S9; less sandy loam with 2.2% humus, pH 7.1, 51°46′2.11″N, 11°9′3.64″E), Sickte 10 (S10; less sandy loam with 1.1% humus, pH 5.9, 52°12′45.2″N, 10°38′20.7 E), Bundesallee (less sandy loam with 1.4% humus, pH 6.2, B; 52°17′57″N, 10°26′14″E), and Elsdorf (E; heavy sandy loam with 1.7% humus, pH 7.2, 50°55′41.74″N, 6°33′56.81″E). The soils were sieved through a 1 mm sieve and 10 g of sieved soil blended with 2 × 20 ml of STW for 1 min at a high speed (Stomacher®80, LAB SYSTEM). The supernatant containing released soil microbes was centrifuged for 5 min at 500 × g to remove soil particles, and sieved through a sterile 5-µm sieve to exclude indigenous nematodes and any remaining larger particles. The flow through was used as microbial suspension for baiting of J2.

For baiting, a sterile 5-µm sieve containing the surface-sterilized nematodes was immersed in 25 ml of soil suspension in a glass jar and incubated overnight at 20 °C on a shaker at 30 rpm. After incubation, nematodes on the sieve were washed with 50 ml of STW to remove loosely attached microbes. The nematodes with attached microbes were pelleted at 1000 x g for 2 min, resuspended in STW and the number of recovered nematodes determined under the microscope. The suspension was plated on R2A media (Merck, Germany) supplemented with cycloheximide (10 mg/l). The plates were incubated for 2 days at 28 °C before isolation of bacterial strains from single colonies.

### Characterization of nematode-attached bacterial isolates

About 10 µl of bacteria lawn was suspended in 100 µl TE buffer. Cells were lysed by adding 100 µl of 50 mM Tris-HCl pH 8.0/50 mM EDTA/0.5% Tween 20/0.5% Triton X-100, containing 200 µg lysozyme, 90 µg proteinase K and 20 µg RNase A. They were incubated at 37 °C for 30 min. This was followed by the addition of 3 M guanidine hydrochloride/20% tween 20 and incubation at 50 °C for 30 min. The DNA from the lysate was captured for 5 min with 200 µl GeneClean Spin Glassmilk (MP Biomedicals). The pelleted Glassmilk was washed twice with 500 µl washing solution (100 mM NaCl/1 mM EDTA/10 mM Tris-HCl, pH 7.5/50% EtOH), and air-dried for 10 min. Finally, the DNA was eluted with 100 µl 10 mM Tris-HCl/0.1 mM EDTA pH 8.0 and separated from the Glassmilk by centrifugation at maximum speed for 2 min (12,000 × g). The supernatant containing the bacterial DNA was stored at −20 °C until use.

Bacterial isolates were compared by BOX-PCR fingerprinting^58^. Briefly, ca. 20 ng of template DNA was added to a 25 µl PCR reaction containing GoTaq Flexi Buffer, 3.75 mM MgCl_2_, 0.2 mM dNTP, 5% w/v DMSO, 0.2 mM primer BOXA1R (5′‐CTA CGG CAA GGC GAC GCT GAC TGA CG‐3′) and 1 U GoTaq Flexi DNA polymerase (Promega). Amplifications were performed in a thermocycler (Mastercycler, Eppendorf) using the following conditions: denaturation step for 7 min at 94 °C, 35 cycles of 1 min at 94 °C, 1 min at 53 °C, and 8 min at 65 °C. The final extension step was 16 min at 65 °C. The PCR products were electrophoresed in 1.5% agarose gel (Roth, Germany) for 3 h at 80 V. The band patterns were visualized by UV transillumination (254 nm) after staining with ethidium bromide and analyzed by GelCompar II (Applied Maths, Sint-Martens-Latem, Belgium).

For strains with a unique BOX fingerprint, a 1.5 kbp long 16S rRNA gene fragment was amplified from the bacterial DNA in a 25 µl PCR reaction containing TrueStart Buffer, 3.75 mM MgCl_2_, 0.2 mM dNTP, 5% w/v DMSO, 0.1 mg/ml BSA, 0.1 mM forward primer U8-27 (AGA GTT TGA TC(AC) TGG CTC AG)^59^, 0.1 mM reverse primer R1494-1514 (CTA CGG(T/C) TAC CTT GTT ACG AC)^60^, and 1 U TrueStart Taq DNA Polymerase (Thermo Scientific). The PCR conditions consisted of the initial denaturation step at 94 °C for 5 min, 30 cycles of 94 °C for 1 min, 56 °C for 1 min, 72 °C for 1 min, and a final elongation step at 72 °C for 10 min. The PCR products were purified using the HighPure PCR Product Purification Kit (Roche Life Sciences) and sequenced using a set of reactions with different primers (Macrogen, Amsterdam, The Netherlands). The forward and reverse sequences were aligned in MEGA7^61^ using ClustalW and blasted against NCBI GenBank.

### Adhesion studies

The 15 different bacterial isolates were tested for their re-attachment to *M*. *hapla* J2. The main cultures of the isolates were grown in liquid LB media at 28 °C overnight, short-spinned for 30 seconds and resuspended in STW. The triplicate glass incubation tubes containing 500 surface-sterilized *M*. *hapla* J2 in 250 µl of bacterial suspension were incubated overnight at room temperature. As a reference, 250 µl of a rifampicin resistant mutant of the isolate i.10 (i.10-rif) was eventually added to each replicate tube. To remove non-attached cells we modified the previously published method^62^. The glass tube-content was transferred to a 1.5 ml microtube and centrifuged at 1000 x g for 1 min at room temperature. The supernatant was removed and centrifugation repeated twice using 1 ml of STW. Finally, 1 ml of STW and 0.2 g of 0.1 mm glass beads were added to each tube. These were vortexed for 10 s, serially diluted and plated on R2A media. The plates were incubated for 2 days at 28 °C to determine the number of CFU per J2.

To compare the bacterial attachment to *M*. *hapla* and *P*. *penetrans*, 500 surface-sterilized juveniles of *M*. *hapla* and *P*. *penetrans* were incubated overnight in 400 µl suspension of bacterial isolates i.27, i.14, i.37, i.44, E1, K6, BS1-2, or the control *E*. *coli* EK5-23. To analyze attachment dynamics, 500 surface-sterilized *M*. *hapla* J2 were incubated for 2, 4, 6, 24, and 48 h in 500 µl of a bacterial suspension containing equal volumes of the isolates K6 (10^8^ CFU/ml) and i.10-rif as a reference (10^6^ CFU/ml). To determine whether different bacterial concentrations would affect the attachment rate, *M*. *hapla* J2 were incubated in 100 µl of three different serial dilutions of the isolates i.10 (10^6^, 10^5^ and 10^4^) and i.20 (10^5^, 10^4^ and 10^3^) overnight. To study the effect of a treatment of the nematode surface coat by detergents on bacterial attachment, 500 surface-sterilized *M*. *hapla* J2 were incubated in 5 ml of 0.25% SDS (sodium dodecyl sulfate), 5 ml of 0.25% CTAB (cetyltrimethylammonium bromide), or 5 ml of STW for 1 hour. The detergents were removed by washing nematodes on sterile 5-µm sieves with STW until the foam disappeared. The J2 were then incubated for 3 hours in the bacterial suspension (250 µl of the isolate K6, containing 10^8^ CFU/ml, and 250 µl of the reference isolate i.10-rif, containing 10^6^ CFU/ml).

### Visualization of the nematode-attached bacteria

Surface-sterilized *M*. *hapla* J2 were incubated in a suspension of the bacterial isolate K6 (10^8^ CFU/ml) for 24 hours. Non-attached bacteria were removed by centrifugation. The J2 with and without attached bacteria were observed using a light microscope Axioskop 2 Plus (Zeiss). Samples were also examined using the environmental scanning electron microscopy (ESEM) mode in a Quanta 250 scanning electron microscope (FEI Deutschland GmbH, Germany). A 10-µl sample was applied to a flat specimen stub (1 cm in diameter and 0.5 cm in height) and placed on the Peltier cooling stage that had been mounted into the chamber before analysis. For signal detection, a gaseous secondary electron detector with a 500 µm pressure-limiting aperture was attached below the pole shoe. Images were collected at 12.5 kV, 5 mm working distance, and 720–740 Pa water vapor pressure at 4 °C.

### Biological effect of nematode-attached bacteria

For *in vitro* assays, the main cultures of the bacterial isolates K6, i.44, BS1-2, BS1-7, E1, or *E*. *coli* EK5-23 were grown in 100 ml of liquid LB media overnight, pelleted at 4000 × g for 10 min and resuspended in STW to obtain an OD_600_ of 0.1. The effect of bacteria on J2 mortality was assessed by incubating 500 surface-sterilized J2 of *M*. *hapla* for 3 days in 1 ml of bacterial suspensions in quadruplicates. The numbers of dead J2 were determined for each isolate and compared to STW control. A J2 was considered dead when the body was completely stretched or did not show any movement for more than 5 s. To study the effects of bacteria on J2 motility, 500 surface-sterilized J2 of *M*. *hapla* were incubated in 2 ml of bacterial suspensions in quadruplicates. After 5 days, the suspensions were transferred to sterile 20-µm sieves placed in 6-well plates and the number of J2 that passed through the sieve was determined the next day.

For the hatching assay, eggs of *M*. *hapla* were extracted from tomato roots using 1% NaOCl and purified by centrifugation in 35% sucrose solution. The surface of the eggs was sterilized using 0.5% NaOCl for 2 min and 1000 eggs were incubated for 5 days in 2 ml of the respective bacterial isolate in quadruplicates. The number of hatched J2 from the eggs was determined and compared to a STW control.

To study the effect of the bacterial isolates K6, i.44, BS1-2, BS1-7 and E1 on J2 invasion into roots, tomato seeds were surface sterilized using 70% ethanol for 1 min and 2.5% NaOCl for 3 min, and planted in pots containing 300 g of a sterile sand-peat moss mixture (10:1). Pots were kept in the greenhouse at 20 ± 2 °C and 16-h photoperiod. Each treatment was replicated ten times and arranged in a randomized block design. After 3 weeks, the top soil layer was removed and the soil was drenched with 15 ml of the respective bacterial suspension or STW as a control. The soil surface was covered with previously removed soil and the pots were watered as needed. Five days after challenging plants with bacterial cells 1000 surface-sterilized *M*. *hapla* J2 were inoculated to each pot. To determine the number of invaded J2 inside the roots, the plants were uprooted seven days post inoculation and the roots stained with acid-fuchsin (Sigma-Aldrich). The effect of each bacterial isolate on J2 invasion was compared to the STW control.

### Statistical analysis

The package SAS 9.4 (SAS Institute Inc., Cary, NC, United States) was used to fit generalized linear models. For count data (number of J2), the procedure GENMOD was applied with Poisson distribution, log link function and specification of a scale parameter (Pearson) to account for overdispersed data. For multiple comparisons to a control, the alpha level was adjusted according to Dunnett.

## Supplementary information

Supplement

## Data Availability

All materials, data and associated protocols are available from the corresponding author on reasonable request. DNA sequences of 16S rRNA genes were deposited in NCBI GenBank with accession numbers MK217825 – MK217841.
