# Supplementary material for: Bacteria isolated from the cuticle of plant-parasitic nematodes attached to and antagonized the root-knot nematode Meloidogyne hapla
Source: Sci Rep. 2019 Aug 7;9:11477. doi: 10.1038/s41598-019-47942-7 (PMC6685954; doi:10.1038/s41598-019-47942-7)
Supplement: Supplementary file 1 — Supplement [file 41598_2019_47942_MOESM1_ESM.pdf]

Supplement to

“Bacteria isolated from the cuticle of plant-parasitic nematodes attached to and antagonized the root-knot nematode *Meloidogyne hapla*“

Olivera Topalović [1], Ahmed Elhady [1,2], Johannes Hallmann [1], Katja R. Richert-Pöggeler [1], Holger Heuer [1*]

[1] Dept. Epidemiology and Pathogen Diagnostics, Julius Kühn-Institut — Federal Research Centre for Cultivated Plants, Braunschweig, Germany

[2] Department of Plant Protection, Faculty of Agriculture, Benha University, Benha, Egypt

*Correspondence: holger.heuer@julius-kuehn.de

Supplementary Table S1. Identification of bacterial strains that were highly attached to J2 of *Meloidogyne hapla* or *Pratylenchus penetrans*, using 16S rRNA gene sequencing.

Isolates	Soil source	GenBank BLAST hit	Accession ID, % identity
S10-12 (Mh)	Sickte (field 10)	*Acinetobacter* sp.	KX_982223.1, 99%
E1 (Mh)	Elsdorf	*Acinetobacter* sp.	KJ_147068.1, 99%
i.44 (Mh)	Geisenheim	*Microbacterium foliorum*	CP_019892.1, 99%
i.47 (Mh)	Geisenheim	*Microbacterium* sp.	CP_018151.1, 99%
BS1-7 (Mh)	Dahnsdorf (field BS1)	*Brevundimonas vesicularis*	MH_144292.1, 99%
S5-5 (Mh)	Quedlinburg (Schlag 5)	*Brevundimonas* sp.	KY_907035.1, 99%
G1 (Mh)	Geisenheim	*Kocuria uropiqioeca*	NR_157676.1, 99%
i.10 (Mh)	Geisenheim	*Microbacterium phyllosphaerae*	NR_025405.1, 99%
K5 (Mh)	Klein Wanzleben	*Micrococcus* sp.	MH_671525.1, 99%
G11 (Mh)	Geisenheim	*Sphingopyxis* sp.	EF_540469.1, 99%
BS1-2 (Mh)	Dahnsdorf (Schlag 2)	*Sphingopyxis* sp.	AB_161684.1, 99%
K6 (Mh)	Klein Wanzleben	*Microbacterium* sp.	MG_266365.1, 99%
K1 (Mh)	Klein Wanzleben	*Staphylococcus equorum*	MF_578770.1, 99%
i.20 (Mh)	Geisenheim	*Microbacterium* sp.	EU_747700.1, 99%
i.14 (Pp)	Bundesallee (bulk soil)	*Microbacterium oxydans*	MF_459692.1,100%
i.27 (Pp)	Bundesallee (maize rhizosphere)	*Pseudomonas protegens*	NR_114749.1, 100%
i.37 (Pp)	Bundesallee (soybean rhizosphere)	*Alcaligenes faecalis*	NR_113606.1, 100%
